# Increased sex ratio in Russia and Cuba after Chernobyl: a radiological hypothesis

**DOI:** 10.1186/1476-069X-12-63

**Published:** 2013-08-15

**Authors:** Hagen Scherb, Ralf Kusmierz, Kristina Voigt

**Affiliations:** 1 Institute of Computational Biology, Helmholtz Zentrum Muenchen, German Research Center for Environmental Health, Neuherberg, Germany

**Keywords:** Food contamination, Food export import, Human secondary sex ratio, Radiation induced genetic effects, Radioactive fallout

## Abstract

**Background:**

The ratio of male to female offspring at birth may be a simple and non-invasive way to monitor the reproductive health of a population. Except in societies where selective abortion skews the sex ratio, approximately 105 boys are born for every 100 girls. Generally, the human sex ratio at birth is remarkably constant in large populations. After the Chernobyl nuclear power plant accident in April 1986, a long lasting significant elevation in the sex ratio has been found in Russia, i.e. more boys or fewer girls compared to expectation were born. Recently, also for Cuba an escalated sex ratio from 1987 onward has been documented and discussed in the scientific literature.

**Presentation of the hypothesis:**

By the end of the eighties of the last century in Cuba as much as about 60% of the food imports were provided by the former Soviet Union. Due to its difficult economic situation, Cuba had neither the necessary insight nor the political strength to circumvent the detrimental genetic effects of imported radioactively contaminated foodstuffs after Chernobyl. We propose that the long term stable sex ratio increase in Cuba is essentially due to ionizing radiation.

**Testing of the hypothesis:**

A synoptic trend analysis of Russian and Cuban annual sex ratios discloses upward jumps in 1987. The estimated jump height from 1986 to 1987 in Russia measures 0.51% with a 95% confidence interval (0.28, 0.75), p value < 0.0001. In Cuba the estimated jump height measures 2.99% (2.39, 3.60), p value < 0.0001. The hypothesis may be tested by reconstruction of imports from the world markets to Cuba and by radiological analyses of remains in Cuba for Cs-137 and Sr-90.

**Implications of the hypothesis:**

If the evidence for the hypothesis is strengthened, there is potential to learn about genetic radiation risks and to prevent similar effects in present and future exposure situations.

## Background

### Sex ratio – a genetic indicator

According to Schull and Neel [1-3], the uniqueness of the human sex ratio at birth as an indicator of genetic health or genetic detriment arises from the fact that maternal chemical or physical mutagenic exposure is expected to produce a sex ratio different from the sex ratio after paternal exposure. Therefore, the ratio of male to female offspring at birth may be a simple and non-invasive way to study and monitor the reproductive status of a population. Among others, environmental and occupational hazards can alter the sex ratio at birth. In a recently published comprehensive review article [4], more than 100 studies were evaluated including several investigations on ionizing radiation and chemicals. Among the occupational exposure studies concerning ionizing radiation, Hama et al. [5] considered 586 male radiologists in Japan. As a group, male radiologists tended to father a lower proportion of boys compared with the control group. Maconochie et al. [6] looked at over 46,000 children born to UK nuclear industry workers and found no statistically significant alterations of the sex ratio. However, in a considerably larger study of 260,060 births to fathers employed at Sellafield, Dickinson et al. [7] reported that those men sired a greater proportion of boys than would be expected. An effect was also observed in fathers with recorded doses exceeding 10 mSv before conception. While this may reflect a true statistical association, it is also possible that it may be a chance finding due to imprecision in the dose estimates and consequent misclassification. Animal experiments shed light on the extreme complexity of radiation induced genetic effects. Irradiation of female mice with fission neutrons by Russel et al. [8] has shown that the length of the period between irradiation and conception has a striking effect on the mutation frequencies seen in the offspring. In conceptions seven weeks after irradiation, mutation frequencies turned out to be relatively high. Havenstein et al. [9] have shown that radiation exposure of spermatogonia entailed a real change in the sex ratio in the rat. Nevertheless, Russell and Havenstein doubt that their positive results received with mice and rats will apply to humans. Neel et al. [10] studied children of parents exposed to atomic bombs in Japan on the basis of revised radiation dose estimates. These revised estimates indicated that humans are less sensitive to genetic effects from radiation than has been assumed on the basis of extrapolations from animal experiments. However, this point of view has been challenged by Vogel [11].

According to Scholte and Sobels [12], one of the few methods available for studying the genetic effects of ionizing radiation in man in sufficiently large populations is the observation of changes in the sex ratio among offspring from irradiated parents. Radiation induced lethal factors of varying degree of dominance on the X chromosome depending on whether an impaired X chromosome is derived from the mother or the father impact the formation and the survival probability of the female zygote, entailing more or less girls at birth, which can also be interpreted as less or more boys, respectively. According to theory [13], Cox found reduced offspring sex ratio (deficit of boys) in irradiated women [14], and James emphasized “ionizing radiation is the only reproductive hazard, which causes men to sire an excess of sons” [15]. In addition to lethal factors on the X chromosome, Scholte and Sobels [12] allude to nondisjunction resulting in X0 genotypes, which are non-viable in man and, thus, may also distort the birth sex ratio. As Down syndrome is a well-known consequence of meiotic nondisjunction, evidence of increased nondisjunction across Europe after Chernobyl is obtained from increased Down syndrome prevalence at birth [16]. Except in societies where selective abortion skews the sex ratio [17-19], approximately 104 to 106 boys are born for every 100 girls. In humans, on the one hand, the sex ratio at birth is essentially constant at the secular population level [20], but on the other hand, considerable variability of the sex ratio may be observed under a variety of specific circumstances. A lot of hypothetical sex ratio determinants and methodological challenges assessing them have been discussed in the literature [21]. However, Steiner [22] points out that proposed determinants showed associations in small samples that could not be replicated in larger populations. This, of course, may be due to insufficient statistical power, i.e., large second kind error probabilities due to small effects or too small study-populations.

## Offspring sex ratio – in atomic bomb survivors and in parents hit by nuclear testing

**Figure 1:**
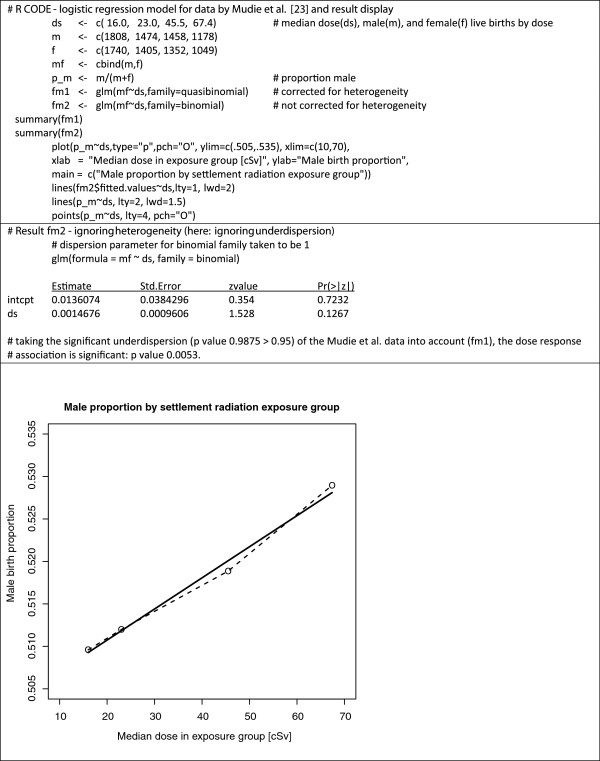
**R code for sample logistic regression, result summary, and graphical display of the Mudie et al. data **[23]**.**

Schull and Neel performed studies in the sex ratio among infants born to survivors of the atomic bombings of Hiroshima and Nagasaki, Japan. The first study published in 1958 [1] revealed significant changes in the sex ratio of these children. The second study [2] still found a small effect in the early post-bomb years, which had apparently disappeared in later years. Schull et al. [2] explained: “One can argue that a small early effect has disappeared or that the original observation had no biological significance”. Mudie et al. [23] studied the sex ratio in the 11,464 offspring of parents with chronic radiation exposure from nuclear testing in Kazakhstan. They conclude: “No significant association was found between radiation exposure level and sex ratio, but some previously suggested demographic factors were positively associated with sex ratio.” However, looking at the tabulated Mudie et al. data, we can see that the sex ratio increases linearly from 1.04 at less than 20 cSv, to 1.05 at 20–40 cSv, to 1.08 at 40–60 cSv, and to 1.12 at more than 60 cSv. See Figure 1 for a sample logistic regression analysis of this data set using the statistical freeware package “R”. For an introduction to logistic regression see [24]. Although the Mudie et al. result was not significant, it is nevertheless consistent with a positive association of the sex ratio with radiation exposure; quite similar in principle to what we have found at the ecological district level in Germany after Chernobyl [25].

## Sex ratio in Europe after Chernobyl

**Figure 2:**
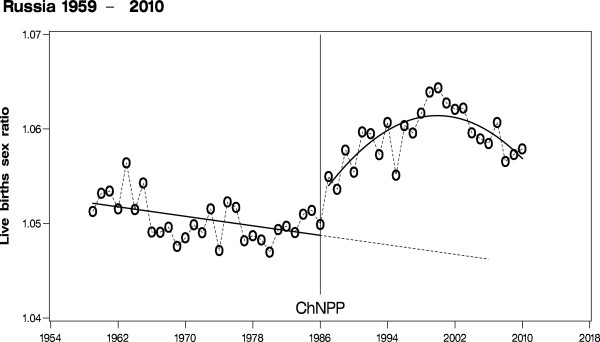
The human secondary sex ratio in the Russian Federation including logistic regression model; ChNPP: Chernobyl Nuclear Power Plant explosion.

Motivated by Schull and Neel’s publication [1] and since we had found increased stillbirths and birth defects after Chernobyl [26,27], we have been investigating the influence of ionizing radiation on the human birth sex ratio for several years. By a pilot study, we assessed the trends in the sex ratio in several selected European countries with emphasis on the Chernobyl Nuclear Power Plant accident [25]. As this study yielded positive results including an ecological dose response association between fallout and the sex ratio, we investigated the behavior of the sex ratio after the atmospheric atomic bomb tests and after Chernobyl more thoroughly for longer time periods and on a global scale. One of the main results was a jump of the sex ratio after Chernobyl in all of Europe, including Russia (Figure 2), and a subsequent trend reversal from 1987 onward. No such similar effect was seen in the less affected USA. This investigation [28] confirmed our opening study [25]. For debate and further findings see [29-32]. Peterka et al. [33] reported a sharply reduced male live birth proportion in November 1986 in the Czech Republic. The decreased male proportion restricted to a single month is in contrast to the long term increased male proportion across Europe. Moreover, replication of the Peterka et al. study with Bavarian data yields an estimate of the male proportion in November 1986 identical to the overall mean. Therefore, the finding by Peterka et al. could not be supported [34].

## Sex ratio in Cuba after Chernobyl

### S. J. Venero Fernandez et al

**Figure 3:**
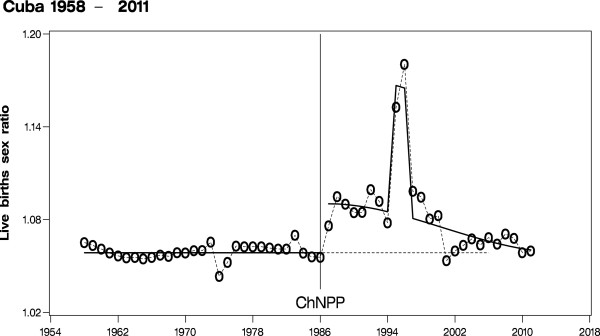
The human secondary sex ratio in the Cuba including logistic regression model adjusted for extreme values in 1995 and 1996; ChNPP: Chernobyl Nuclear Power Plant explosion.

An intriguing new example of an escalated sex ratio after Chernobyl has been published in the American Journal of Epidemiology by Cuban scientists [35]. In Cuba, the sex ratio is subject to a strong uptick immediately after Chernobyl in the year 1987 (Figure 3). Moreover, this jump in the sex ratio is followed by a long-lasting elevated trend up to the year 2000 when the Cuban sex ratio approaches 1.06 – 1.07, which are nearly pre-Chernobyl values. Contrary to the Trivers-Willard hypothesis postulating decreasing sex ratios during economic hardship [36], Venero Fernandez et al. [35] try to explain the striking sex ratio increase in Cuba by a sociological aspect, namely by the economic depression in Cuba (‘Special Period’), which started in 1991 after the dissolution of the Soviet Union and the COMECON. However, the strong increase from the stable sex ratio of 1.0585 in the 29-years 1958 – 1986 to the escalated average sex ratio of 1.0864 (1.0785, 1.0944), p value < 0.0001 in the 4-years period 1987 – 1990 can hardly be explained neither by chance nor by economic depression. Chance can be excluded as this jump from the level in 1958 – 1986 to the level in 1987 – 1990 measures more than 5 standard errors and economic depression can be excluded as its onset occurred only 4 years after the sex ratio jump, see the GDP curve in Figure 2 in [35].

### A. J. Wilcox and D. D. Baird

Together with the publication by Cuban scientists, an invited commentary by two American scientists appeared in the same issue of the American Journal of Epidemiology. Wilcox and Baird question the importance of the sex ratio as an environmental health indicator and try to explain the strong increase in the sex ratio in Cuba by sex selective abortions [37]. Abortions in Cuba have been described in the scientific literature [38] but not gender-specific ones [39]. Therefore, the alternative explanation offered by Wilcox and Baird, sex selective abortion, is implausible as this practice is unusual in Cuba. Also, Wilcox and Baird do not address the question as to why sex selective abortion starts in 1987, which is not congruent with the ‘Special Period’ that began only in or after 1990.

### L. Simpson

Simpson [40] attempts to explain the effect by a technical artifact of the data recording procedure, possibly caused by and acting from the ending of the former Soviet Union in 1990: “Russia’s breaking of economic trade agreements with Cuba in 1990 was followed by a tightening embargo on trade from the US government … As one specific example, there were insufficient funds to continue to import a gummed 2-page form that accurately replicated writing onto a copy. This form had been used to record birth details in hospitals, where over 99% of births in Cuba occurred throughout this period”. Again, Simpson oversees the significant uptick of the sex ratio in 1987 together with the even stronger increases in 1988 through 1990, clearly emerging before the Soviet breakdown. Quantifying this peculiar and stable 4-year increase from 1987 – 1990 yields a sex ratio ratio (or better sex odds ratio) of 1.0263 (1.0209, 1.0318), p value < 0.0001. Therefore, Simpson’s explanation does not apply to the period from 1987 through 1990, and thus his explanation may perhaps only partly account for the escalated sex ratio in Cuba from 1991 onward. Also, Simpson does not make sufficiently clear why a presumable random noise imposed on the recordings of the births’ sexes should be biased in favor of boys; one would rather expect non-differential misclassification instead. Eventually, one might speculate that Simpson’s explanation is to the point in principle, but only for the years 1995/1996. However, this is not important for our hypothesis as we focus on the years 1987/1988, and Simpson did not restrict his argument to 1995/1996. The adjustment for the years 1995 and 1996 in our Cuban sex ratio trend model (Figure 3) is equivalent to excluding those years as outliers. Therefore, excluding these outliers would not change our effect estimates, confidence limits, and p-values. Consequently, our inference from the Cuban sex ratio data is independent from those outliers, and is thus somewhat conservative. In summary, no convincing explanation of the strong and transient sex ratio increase in Cuba from 1987 to 2000 has been offered in the literature as yet, neither by the authors themselves nor by the annotators.

## Presentation of the hypothesis

There is no denying the fact that a strong and highly significant increase in the human sex ratio at birth in Cuba immediately after 1986 exists, and 1986 was the year of the Chernobyl accident. A sex ratio increase after 1986 also holds true for all of Europe, and in particular for single southern and eastern European countries, among them Russia [28,32]. The question arises whether any more or less smooth social, political, economic, etc. factor, could entail such an abrupt consequence across Europe and in Cuba simultaneously. Therefore, we hypothesize that the mechanism might be a direct bio-physical one that acts synchronously in Europe, in Russia, and in Cuba from 1987 onward. It must not be overseen that according to the Trivers-Willard hypothesis [36], the effect in Cuba is in the wrong direction. Economic depression would lead to a decrease not an increase in the sex ratio. On the other hand, according to James [15], radiation is the only known reproductive hazard that increases the sex ratio. From this perspective, the previous explanation attempts are not convincing. Therefore, we are of the opinion that there is a direct effect of radioactively contaminated food and possibly feeding stuff exported from the former Soviet Union or from other Chernobyl affected European or Asian countries to Cuba. During the eighties of the last century in Cuba more than 50% of the food imports were provided by the former USSR. In the Seattle Post-Intelligencer, Buncombe [41] explained: "Cuba's economy was extraordinarily reliant on subsidies from its political older brother, the Soviet Union. Its agriculture was designed with one aim in mind – namely to produce as much sugar cane as possible, which the Soviets bought at more than five times the market price, in addition to purchasing 95 percent of its citrus crop and 73 percent of its nickel. In exchange, the Soviets provided Cuba with 63 percent of its food imports and 90 percent of its petrol. Such a relationship made Cuba extraordinarily vulnerable". From statistics published by the Food and Agriculture Organization of the United Nations (FAO, http://faostat.fao.org/), we can see for example that in the period 1986 – 1989 evaporated milk in the range of over 100,000 tons was imported in Cuba from the former USSR (Table 1). Interestingly, the amount of imported milk doubled just in 1987, however, at half the price compared to the remaining years.

**Table 1:** **Evaporated whole milk exports from the former USSR to Cuba in the period 1986 – 1989, see**http://faostat.fao.org/

**Reporter**	**Partner**	**Item**	**Element**	**Year**	**Units**	**Value**	**Flag**
USSR	Cuba	Milk whole Evp	Export quantity	1986	Tonnes	21086	Official data
USSR	Cuba	Milk whole Evp	Export quantity	1987	Tonnes	55543	Official data
USSR	Cuba	Milk whole Evp	Export quantity	1988	Tonnes	21378	Official data
USSR	Cuba	Milk whole Evp	Export quantity	1989	Tonnes	20624	Official data
USSR	Cuba	Milk whole Evp	Export value	1986	1000 US$	20996	Official data
USSR	Cuba	Milk whole Evp	Export value	1987	1000 US$	24588	Official data
USSR	Cuba	Milk Whole Evp	Export value	1988	1000 US$	24738	Official data
USSR	Cuba	Milk whole Evp	Export value	1989	1000 US$	22855	Official data

Ross [42] explained the difficult food supply situation in Cuba during the so-called "Periodo Especial", that is to say in the early years of the 1990s. Cuba had lost Soviet and Eastern Bloc trade preferences and per capita caloric consumption had fallen about 20%. Conversely, this means that the equivalent amount of food representing 20% of per capita caloric consumption can be attributed to imports from the Soviet Union before the crisis, especially from 1987 to 1990. During the ‘Special Period’, imported food has been a relevant factor of feeding Cuba’s population. Imports of dairy products, corn, wheat, wheat flour, fed grains and barley declined in the mid-1990s. We, therefore, presume that Cuba’s imported food and probably feed products before the onset of the crisis were contaminated with radioactive elements from affected European and Asian countries after the Chernobyl accident. Comparison of Figure 1 and Figure 2 indicates that the Cuban effect, although much stronger than the overall Russian effect, seems to vanish somewhat earlier than the effect in Russia. This fits the assumption that ‘only’ imported food was transitionally contaminated and not the whole surface of Cuba. It is even conceivable that contaminated produce found boosted its way to Cuba simply because it was cheaper and Cuba underwent difficult economic conditions, which prevented it from taking effective counter measures to protect its people. The causal interpretation by Venero Fernandez et al. [35]: “These data suggest that, in Cuba, contrary to the Trivers-Willard hypothesis [36], the human population responded to conditions of scarcity by increasing the ratio of males to females at live birth” goes along with our view on this problem, however, in a more concrete biological sense: We are of the opinion that radioactively contaminated human food and probably animal feed induced the increase in the human sex ratio at birth in Cuba after Chernobyl. Unlike other countries [43] and due to political constraints as well as its overall poor position, Cuba as a nation had not the necessary economic and political strength to circumvent the threat of contaminated consumer products after Chernobyl by imposing safe control measures on imports from abroad.

## Testing of the hypothesis

### Synoptic analysis of Russian and Cuban secular sex ratio trends

We compare the sex ratio trends of Cuba (1958 – 2011) and Russia (1959 – 2010) and quantify pertinent effect-parameters of those trends, especially the jumps in 1987. The relevant annual births figures by gender are presented in Table 2 (for the original data sources see: http://www.one.cu/anuariodemografico2011.htm, http://data.euro.who.int/hfadb/, and http://www.mortality.org). Sex ratio in Russia follows an overall linear decline from 1959 to 1986 with a reduction per 10 years of 0.12% (0.04, 0.20), p value 0.0021 (Figure 2). We may estimate a significant jump of the sex ratio from 1986 to 1987 of 0.51% (0.28, 0.75), p value < 0.0001. From 1987 onward, there is a long-term sex ratio increase to maximum values in 1999/2000 of nearly 1.065 and a subsequent decline after the year 2000. A parsimonious model for the partial Russian sex ratio trend after Chernobyl is a 2^nd^ degree polynomial, i.e. a parabola with p value < 0.0001. If the decline starting in 2000 will continue linearly and undisturbed, the Russian sex ratio is to resume normal pre Chernobyl values near 1.05 beyond the year 2020. Sex ratio in Cuba from 1958 to 1986 follows an essentially constant trend with no strong overall upward or downward tendency before Chernobyl (Figure 3). In Cuba, we may estimate a jump in 1987 of 2.99%; (2.39, 3.60), p value < 0.0001, which is six times the jump estimate of the Russian sex ratio in 1987. Moreover, there are still even stronger increases in Cuba in 1995 and 1996 exceeding a sex ratio of 1.15. A well-fitting, however less parsimonious model for the partial Cuban sex ratio trend after Chernobyl consists of a 3^rd^ degree polynomial adjusted for the extreme values in 1995 and 1996. This model approaches nearly normal pre-Chernobyl values of 1.06 around the year 2010. It is, therefore, quite obvious that in Cuba and Russia the sex ratio trends that had existed before the Chernobyl accident are markedly disturbed immediately after Chernobyl albeit the temporal patterns of the sex ratio changes as well as the maximum values taken on differ considerably between the two countries.

**Table 2:** Annual live births by gender and sex ratio for Cuba and Russia

**Year**	**Cuba**				**Russian Federation**			
	**Total**	**Male**	**Female**	**Sex ratio**	**Total**	**Male**	**Female**	**Sex ratio**
1958	176510	91040	85470	1.0652				
1959	191207	98538	92669	1.0633	2796228	1433060	1363168	1.0513
1960	211620	108940	102680	1.0610	2782353	1427225	1355128	1.0532
1961	231811	119194	112617	1.0584	2662135	1365700	1296435	1.0534
1962	249113	127982	121131	1.0566	2482539	1272461	1210078	1.0516
1963	260224	133615	126609	1.0553	2331505	1197738	1133767	1.0564
1964	266554	136880	129674	1.0556	2121994	1087619	1034375	1.0515
1965	267611	137361	130250	1.0546	1990520	1021560	968960	1.0543
1966	264022	135580	128442	1.0556	1957403	1002152	955251	1.0491
1967	257942	132550	125392	1.0571	1851041	947686	903355	1.0491
1968	251857	129376	122481	1.0563	1816509	930239	886270	1.0496
1969	246005	126506	119499	1.0586	1847592	945265	902327	1.0476
1970	237019	121875	115144	1.0585	1903713	974392	929321	1.0485
1971	256014	131733	124281	1.0600	1974637	1011337	963300	1.0499
1972	247997	127610	120387	1.0600	2014638	1031422	983216	1.0490
1973	226005	116584	109421	1.0655	1994621	1022369	972252	1.0515
1974	203066	103687	99379	1.0433	2079812	1063857	1015955	1.0471
1975	192941	98933	94008	1.0524	2106147	1079901	1026246	1.0523
1976	187555	96637	90918	1.0629	2146711	1100411	1046300	1.0517
1977	168960	87039	81921	1.0625	2156724	1103729	1052995	1.0482
1978	148249	76369	71880	1.0625	2179030	1115420	1063610	1.0487
1979	143551	73949	69602	1.0625	2178542	1114937	1063605	1.0483
1980	136900	70496	66404	1.0616	2202779	1126666	1076113	1.0470
1981	136211	70120	66091	1.0610	2236608	1145239	1091369	1.0494
1982	159759	82242	77517	1.0610	2328044	1192252	1135792	1.0497
1983	165284	85433	79851	1.0699	2478322	1268820	1209502	1.0490
1984	166281	85498	80783	1.0584	2409614	1234760	1174854	1.0510
1985	182067	93511	88556	1.0560	2375147	1217322	1157825	1.0514
1986	166049	85274	80775	1.0557	2485915	1273213	1212702	1.0499
1987	179477	93023	86454	1.0760	2499974	1283425	1216549	1.0550
1988	187911	98210	89701	1.0949	2348494	1204907	1143587	1.0536
1989	184891	96428	88463	1.0900	2160559	1110602	1049957	1.0578
1990	186658	97113	89545	1.0845	1988858	1021248	967610	1.0554
1991	173896	90482	83414	1.0847	1794626	923319	871307	1.0597
1992	157349	82399	74950	1.0994	1587644	816757	770887	1.0595
1993	152238	79459	72779	1.0918	1378983	708689	670294	1.0573
1994	147265	76394	70871	1.0779	1408159	724818	683341	1.0607
1995	147170	78803	68367	1.1526	1363806	700191	663615	1.0551
1996	140276	75941	64335	1.1804	1304638	671430	633208	1.0604
1997	152681	79917	72764	1.0983	1259943	648195	611748	1.0596
1998	151080	78948	72132	1.0945	1283292	660842	622450	1.0617
1999	150785	78308	72477	1.0805	1214689	626149	588540	1.0639
2000	143528	74610	68918	1.0826	1266800	653146	613654	1.0644
2001	138718	71166	67552	1.0535	1311604	675750	635854	1.0627
2002	141276	72686	68590	1.0597	1396967	719511	677456	1.0621
2003	136795	70500	66295	1.0634	1477301	760934	716367	1.0622
2004	127192	65674	61518	1.0676	1502477	772973	729504	1.0596
2005	120716	62219	58497	1.0636	1457376	749554	707822	1.0590
2006	111323	57502	53821	1.0684	1479637	760831	718806	1.0585
2007	112472	57984	54488	1.0642	1610122	828772	781350	1.0607
2008	122569	63378	59191	1.0707	1713947	880543	833404	1.0566
2009	130036	67153	62883	1.0679	1761687	905380	856307	1.0573
2010	127746	65692	62054	1.0586	1788948	919639	869309	1.0579
2011	133067	68464	64603	1.0598				

### Contaminated food on the world markets after Chernobyl

The fact that contaminated food was in transit on the world markets [43] is documented especially for Mexico and Brazil where thousands of tons of contaminated milk powder had to be confiscated after the detection of violations of legal contamination limits for Cs-137. In 1988 in Mexico, the state National Company of People’s Subsistence (CONASUPO) distributed 2,436 tons of milk powder contaminated with Cs-137 after Chernobyl. The company was able to recall or otherwise account for 1,497 tons, and the whereabouts of the rest of the milk powder is unknown [44]. In 1987 in Brazil, import of powdered milk from seven European countries had to be stopped after its Cs-137 contamination due to the Chernobyl accident became known and large amounts of milk powder had already been bought by consumers [45]. We are not aware of any comparable counter measures taken in Cuba to protect people from imported Chernobyl contaminated products. This might be explained in general by the close political connection of Cuba to the Soviet Union at that time, and, in particular, by the intent to build a number of nuclear power plants in Cuba with the help of the USSR to overcome the Cuban dependence on imported oil [46]. That radioactively contaminated food, animal feed, and general consumer products were imported to Cuba can be tested in two ways: firstly, by reconstruction of export/import pathways from Chernobyl affected countries to Cuba in analogy to our Table 1, and secondly, by radiological analyses of possible general remains for Cs-137, and teeth of children and bones of deceased for Sr-90. The radioactive Cs-137 and Sr-90 isotopes have sufficiently long half-lives of approximately 30 years that makes them suited for that purpose.

### Reasoning by analogy

There have been positive epidemiological findings after Chernobyl [16,26,27,47,48]. Therefore, our hypothesis can be tested by scrutinizing Cuban public health statistics for increases after 1986: e.g. stillbirths, perinatal mortality, and infant deaths including corresponding sex ratios. Historical hospital records may reflect increases in disease frequencies: e.g. cancer, diabetes, and heart diseases. Finally, children’s hospitals may have recorded data on the occurrence of chromosome anomalies and birth defects: e.g. Down syndrome, malformation of the heart, and cleft lip and palate.

### Limitations of the hypothesis

One of the major limitations of the hypothesis and its testability is of course the long time period of now 27 years that have passed since the Chernobyl accident. It may prove difficult if not impossible to retrospectively throw light on imports to Cuba. Not to speak of the concrete estimation of the amounts of relevant produce from contaminated parts of Europe and Asia actually processed and consumed in Cuba. Also, it may be difficult to distinguish between more or less affected groups in the Cuban population. Did those who hypothetically ate the most contaminated food have the highest sex ratios among their offspring? This question cannot be answered by the as yet published highly aggregated data. It can possibly be answered if historical regional gender specific birth statistics and regional consumption statistics were available and could be linked appropriately. Another major limitation is the general lack of firm evidence that ionizing radiation increases the human sex ratio. It is even possible that certain kinds of radiation exposures decrease the sex ratio or act neutral on gender. Detailed animal experiments have clearly shown the enormous complexity of the diverse ionizing radiation exposures and mutational outcomes [8,9,11,13]. The biologic, genetic, and social details in which way mankind sustains a stable gender proportion are largely unknown.

## Implications of the hypothesis

If the evidence for the hypothesis can be strengthened by appropriate investigations, this would corroborate similar findings in Europe and Asia. Since in contrast to Europe, the Cuban surface was not contaminated by Chernobyl fallout, the effect must essentially be due to internal radiation, i.e. so called internal emitters, following intake of radioactively contaminated food. This special situation could help to better understand etiologic pathways from food contamination to radiation induced genetic effects. Also, the hypothesis if corroborated would weaken the prevailing opinion, e.g. held by UNSCEAR [49], that radiation induced genetic effects have yet to be detected in humans. If the hypothesis can be confirmed, the Cuban experience dealt with in this paper could be a warning with regard to Fukushima and the unresolved problem of the now existing huge amount of radioactive waste worldwide.

## Abbreviations

ChNPP: Chernobyl Nuclear Power Plant; COMECON: Council for Mutual Economic Assistance; CONASUPO: Compañía Nacional de Subsistencias Populares; Cs: Cesium; cSv: 1/100 sievert; FAO: Food and Agriculture Organization; GDP: Gross domestic product; Sr: Strontium; UNSCEAR: United Nations Scientific Committee on the Effects of Atomic Radiation; USSR: Union of Soviet Socialist Republics; X: X chromosome; Y: Y chromosome.

## Competing interests

The authors declare that they have no competing interests.

## Authors’ contributions

KV encountered the publications on increased sex ratio in Cuba, had the idea to publish a comment or a hypothesis, and contributed to the writing of the paper. RK investigated the FAO statistics, further related statistics, provided Table 1, and also contributed to the writing of the paper. HS conducted the statistical analyses and wrote the paper. All authors read and approved the final version of the manuscript.
